# Optimization and Evaluation of Pretreatment Method for sp-ICP-MS to Reveal the Distribution of Silver Nanoparticles in the Body

**DOI:** 10.1186/s11671-019-3016-9

**Published:** 2019-05-28

**Authors:** Takuya Ishizaka, Kazuya Nagano, Ikkei Tasaki, Hong Tao, Jian-Qing Gao, Kazuo Harada, Kazumasa Hirata, Shigeru Saito, Hirofumi Tsujino, Kazuma Higashisaka, Yasuo Tsutsumi

**Affiliations:** 1 Graduate School of Pharmaceutical Sciences, Osaka University, 1-6 Yamadaoka, Suita, Osaka, 565-0871 Japan; 2 Graduate School of Medicine, Osaka University, 2-2 Yamadaoka, Suita, Osaka, 565-0871 Japan; 3 Institute of Pharmaceutics, College of Pharmaceutical Sciences, Zhejiang University, 866 Yuhangtang Road, Hangzhou, 310058 People’s Republic of China; 4 Graduate School of Medicine and Pharmaceutical Science, University of Toyama, 2630, Sugitani, Toyama-shi, Toyama, 930-0194 Japan; 5 Toyama University Hospital, University of Toyama, 2630, Sugitani, Toyama-shi, Toyama, 930-0194 Japan; 6 The Center for Advanced Medical Engineering and Informatics, Osaka University, 1-6, Yamadaoka, Suita, Osaka, 565-0871 Japan

**Keywords:** Single particle inductively coupled plasma-mass spectrometry, Engineered nanoparticles, Silver nanoparticles, Pretreatment of animal tissues, Risk evaluation

## Abstract

The prevalent use of engineered nanoparticles (ENPs) has increased our exposure to these particles. The current available analytical techniques fail to simultaneously quantify and analyze the physical properties of ENPs in biological tissues. Therefore, new methods are required to evaluate the exposure conditions to ENPs. Single particle inductively coupled plasma-mass spectrometry (sp-ICP-MS) is an attractive approach that can perform quantitative and qualitative analyses of ENPs. However, the application of this approach for biological samples is limited because of the lack of pretreatment methods for effectively recovering ENPs from biological tissues. In this study, we evaluated various pretreatment methods and identified the optimal pretreatment conditions for sp-ICP-MS analyses of ENPs in biological tissues using silver nanoparticles (nAg) as a model. We screened five reagents as pretreatment solvents (sodium hydroxide, tetramethylammonium hydroxide, nitric acid, hydrochloric acid, and proteinase K). Our results showed that treatment with sodium hydroxide was optimal for detecting nAg in the mouse liver. Moreover, this pretreatment method can be applied to other organs, such as the heart, lung, spleen, and kidney. Finally, we evaluated the applicability of this method by analyzing the quantity and physical properties of silver in the mouse blood and liver, after intravenous administration of nAg or silver ion. Our sp-ICP-MS method revealed that nAg administered into the blood was partially ionized and tended to be distributed in the particle form (approximately 80%) in the liver and in ionic form (approximately 95%) in the blood. In conclusion, we optimized pretreatment strategies for sp-ICP-MS evaluation of ENPs in biological tissues and demonstrated its applicability by evaluating the changes in the physical properties of nAg in the liver and blood. We also showed that partial changes from the particle form to the ionic form of nAg influences their kinetics and distribution when administered to mice.

## Introduction

Recent progress in nanotechnology has accelerated the development of engineered nanoparticles (ENPs) that are smaller than 100 nm. Because of their beneficial properties such as enhanced tissue permeability and surface reaction compared to other micro or larger sized materials, ENPs are widely used in various products including cosmetics, foods, and medicines [1, 2]. For example, silver nanoparticles (nAg), one of the most common ENPs, are used in antibiotics because of their steady release of Ag^+^. Moreover, they are utilized as conductive materials in printed electronics technology [3]. In contrast, the unique physicochemical properties associated with the small particle size of nAg can be hazardous. It is known that these particles can disrupt the blood-brain barrier and induce inflammation [4]. The increased use of ENPs in daily use products has exposed humans to various types of ENPs. Their continued use should be evaluated to determine their safety [2, 3].

In order to ensure safety, it is indispensable to understand the “risk” of ENPs, which is the integrative concept of “hazard” (potential toxicity) and “exposure condition.” While hazards of ENPs have been analyzed worldwide, few studies have examined the exposure situations to ENPs [5]. Furthermore, it was recently reported that the intracellular distribution of nAg incorporated into cultured cells differs from that of Ag^+^ [6] and that Ag^+^ particulates in mouse tissue [7]. Therefore, it is necessary to evaluate their physical properties, such as particle size and distinguish between particles and ions in the body [3, 6–8].

Using currently available analytical technology, it is challenging to quantitatively analyze the physical properties of ENPs in the body. Inductively coupled plasma-mass spectrometry (ICP-MS) is suitable for quantitative analyses but not for physical property analyses, as all targets such as ions and particles cannot be distinguished during quantification. In contrast, transmission electron microscopy (TEM) is suitable for analyzing the physical properties but not for quantifying ENPs, as only a part of the tissue is observed. Therefore, a novel method is needed for simultaneous physical property analyses and quantitative analyses of ENPs to study their biotransformation.

Single particle-ICP-MS (sp-ICP-MS), which is based on ICP-MS introduces one or no particles into the analyzer per dwell time and is an attractive method for determining particle sizes by analyzing peak intensity and particle concentrations by analyzing peak rates. Particles and ions can be distinguished by analyzing peak signals and background signals [9]. A few previous studies reported the use of sp-ICP-MS for the quantification and physical property analyses of ENPs [10, 11].

However, most of these studies used sp-ICP-MS to analyze environmental water or commercial products containing ENPs [10, 11] and a few studies adopted sp-ICP-MS for biological tissues. Additionally, these studies pretreated the tissues by proteinase K digestion or with tetramethyl ammonium hydroxide (TMAH) to solubilize the protein and lipid matrices. As different reagents have different solubilizing properties, variations in pretreatment methods may influence the recovery rate of ENPs distributed in the tissues. Furthermore, it is important that the pretreatment method does not affect the size or ionic properties of ENPs and efficiently recovers the ENPs distributed in the tissues.

In this study, we evaluated and optimized different pretreatment methods for sp-ICP-MS in biological samples to determine the quantity and physical properties of ENPs in the body using nAg as model ENPs.

## Materials and Methods

### ENPs

The 30, 70, and 100 nm “Biopure” nAg (nAg30, nAg70, and nAg100) were obtained from nanoComposix (San Diego, CA, USA). RM8013 was used as a standard for calculating transport efficiency and was obtained from the National Institute of Standards and Technology (Gaithersburg, MD, USA). Each type of ENPs was sonicated for 10 min prior to use.

### Reagents

Solutions of 0.1 mol/L sodium hydroxide (NaOH), 25% TMAH, 30% hydrochloric acid (HCl), and proteinase K were obtained from Wako (Osaka, Japan). A solution of 70% nitric acid (HNO_3_) was obtained from Kanto Kagaku (Tokyo, Japan).

### Animals

BALB/c mice (female, 6 weeks) were purchased from Japan SLC (Shizuoka, Japan). Mice were housed in a room with a 12-h light/dark cycle (lights on at 8:00 and lights off at 20:00). Food and water were provided ad libitum. The experimental protocols adhered to the ethical guidelines of Osaka University, Japan.

### Measuring Particle Size Distributions by Dynamic Light Scattering

nAg was diluted in milliQ water to a final silver (Ag) concentration of 10 μg/mL. Next, the size and zeta capillary cell (Malvern Instruments, Malvern, UK) was filled with 1 mL of the solution to measure the particle distribution and mean diameter with a Zetasizer Nano-ZS (Malvern Instruments).

### Measuring Gross Mass of Ag

To measure the total Ag concentration in the samples, an Agilent 7700x (Agilent Technologies, Santa Clara, CA, USA) was used. The analysis conditions were RF power 1550 W, carrier gas 1.05 L/min Ar, and dwell time 100 ms. Measurements were repeated three times in MS mode. An internal standard method was used, and rhodium was used as an internal standard for Ag. Target elements of ICP-MS analyses were ^103^Rh and ^107^Ag. Ag and rhodium standard solutions were obtained from Wako (Osaka, Japan).

### Analysis of sp-ICP-MS and Its Calculation

For sp-ICP-MS analysis, we used an Agilent 7700x (Agilent Technologies; Santa Clara, CA, USA) similar to the analysis of total Ag. The analysis conditions were as follows: RF power 1550 W, carrier gas 1.05 L/min Ar, dwell time 10 ms, and analysis time 30 s. In order to calculate the particle size, single particle calculation tools published by RIKILT was used [12].

### Critical Particle Concentration for sp-ICP-MS

The stock nAg solution concentration was 1.0 mg/mL, which was used to prepare 2000, 800, 700, and 600 pg/mL solutions. Each of these solutions was then serially diluted 10 times to obtain 40 different nAg solutions. We determined the particle concentrations of these 40 samples by sp-ICP-MS.

### Optimization of Pretreatment Methods for Mouse Liver

The livers collected from the mice were mixed with phosphate-buffered saline (PBS) (*w*/*v* ratio of 1:10) and then homogenized. The homogenate was mixed with 100 ng/mL nAg solution. The mixture was then treated with one of the following reagents at a *v*/*v* ratio of 1:1—0.1 mol/L NaOH solution, 25% TMAH, 30% HCl, or proteinase K solution (10 U/mL proteinase K, 0.01 M Tris-HCl, 0.01 M EDTA, and 0.5% SDS). The samples were incubated for 3 h at 37 °C and the residues were collected and weighed. Supernatants were diluted 500-fold and analyzed by sp-ICP-MS.

### Versatility Evaluation of NaOH Pretreatment in Various Organs

The hearts, lungs, spleens, and kidneys collected from the mice were mixed with PBS (*w*/*v* ratio of 1:10), homogenized, and mixed with 100 nm/mL nAg. Next, 1 mol/L NaOH solution at a *v*/*v* ratio of 1:1 was added and incubated for 3 h at 37 °C. After incubation, the residues were collected and weighed. The supernatants were diluted 500 times and analyzed by sp-ICP-MS.

### Evaluation of Quantity and Physical Properties of nAg100 and Ag^+^ in Mice After Single Intravenous Administration

For intravenous administration, nAg100 and AgNO_3_ were diluted to 0.25 mg/mL (as Ag^+^) with 5% glucose solution. BALB/c mice were intravenously administered with nAg100 (1.5 or 0.75 mg/kg), AgNO_3_ (1.5 or 0.75 mg/kg as Ag^+^), or 5% glucose solution (control). After 24 h, the blood and livers of the sacrificed mice were collected. The livers were mixed with PBS (*w*/*v* ratio of 1:10) and homogenized. The blood and liver homogenates were mixed with TMAH solution (*v*/*v* ratio of 1:1) and with NaOH solution (*v*/*v* ratio of 1:1), respectively. These samples were analyzed by ICP-MS to measure the Ag concentrations and by sp-ICP-MS to evaluate the physical properties, such as particle size and distinction between particles and ions.

## Results and Discussions

### Optimization of Particle Detection by sp-ICP-MS

In sp-ICP-MS analysis, it is important to introduce one or no particle into the detector per dwell time. If multiple particles are introduced into the detector over the dwell time, the gross mass of multiple particles is regarded as the mass of a single particle [13]. Therefore, samples must be sufficiently diluted for sp-ICP-MS analysis. In contrast, sp-ICP-MS analysis of a sample with a very low concentration of ENPs leads to inaccurate particle distribution and size data.

**Fig. 1:**
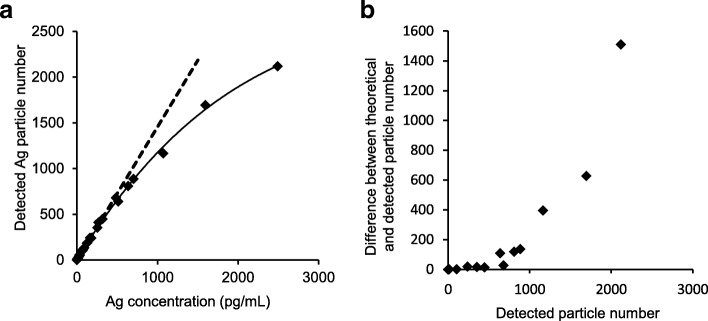
Determination of optimal particle number per dwell time for accurate sp-ICP-MS analysis. A series of nAg solutions (600 fg/mL to 2,500 pg/mL) were analyzed by sp-ICP-MS. **a** To determine the relationship between the concentration of nAg100 and the number of detected particles, a curve for the detected particles (solid line) the theoretical values (dotted line) were plotted. **b** The number of detected particles subtracted from the theoretical value was plotted in the vertical axis to determine the optimal particle number. Each point is the result of a single trial (*n* = 1)

To determine the relationships between the concentration of nAg100 and number of detected particles, we serially diluted the nAg100 stock solutions for evaluation by sp-ICP-MS. The result showed that the number of detected particles increased theoretically and linearly in the relatively lower Ag concentration region. In contrast, at relatively higher Ag concentrations, the number of detected particles was lower than the theoretical value (Fig. 1a). This data indicated that at higher Ag concentrations, multiple particles tend to be introduced into the detector during each dwell time, resulting in the overestimation of the particle size. Thus, it is necessary to determine the largest number of detected particles that do not differ from the theoretical value to accurately assess particle sizes. Next, we subtracted the number of detected particles from the theoretical value and plotted the difference as the vertical axis. The results indicated that discrepancies in size estimation occurred when the number of detected particles was > 500. This suggests that it is necessary to detect ≤ 500 particles during the analysis time (Fig. 1b). Although these data were obtained in a single trial, repeating the experiment showed the same results (data not shown).

In order to validate the analysis conditions, we diluted nAg with various diameters (nAg30, nAg70, nAg100) to detect < 500 particles per analysis time and evaluated their diameters. The sp-ICP-MS analysis indicated that the primary diameters of nAg30, nAg70, and nAg100 were 30.0 ± 1.2, 65.1 ± 0.6, and 97.4 ± 0.6, respectively. Moreover, the hydrodynamic diameters determined by dynamic light scattering (DLS) were 36.4 ± 1.6, 70.6 ± 1.7, and 101 ± 1.0, respectively, these values are similar to those estimated by sp-ICP-MS. These findings suggest that the sp-ICP-MS conditions were appropriate for measuring the diameters of nanoparticles of various sizes.

### Optimization of Pretreatment Methods for Detecting nAg in Mouse Liver Tissue

To quantify and determine the physical properties of ENPs in the body, it is necessary to completely lyse the tissues. Furthermore, it is essential to efficiently recover the particles and ions distributed in the body without inducing any physical or chemical changes in the particles. We tested five solubilizing reagents, NaOH, TMAH, HNO_3_, HCl, or proteinase K, and analyzed the quantity and physical properties by sp-ICP-MS to optimize the pretreatment strategies using the liver as a model [14–18].

**Fig. 2:**
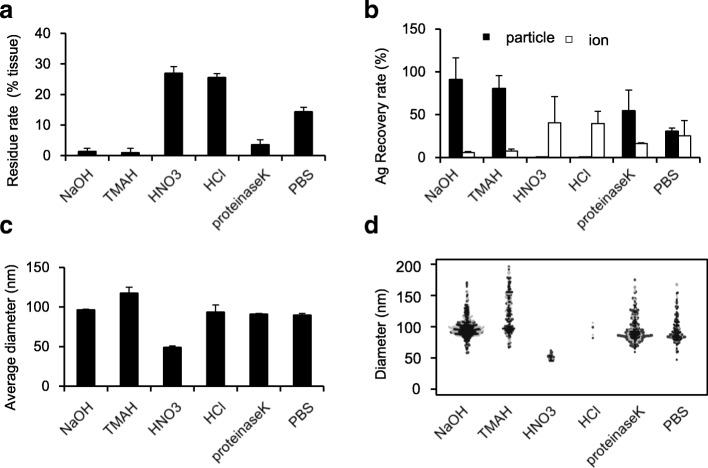
NaOH pretreatment is the optimal method for detecting nAg100 in mouse liver. Five solubilizing reagents were screened as pretreatment solvents to lyse the tissues (NaOH, TMAH, HNO_3_, HCl, and proteinase K). The liver homogenate was mixed with nAg100 solution to obtain a final Ag concentration of 100 ng/mL and treated with each solubilizing reagent at 37 °C. After 3 h, **a** residue rates in each group as an indicator of tissue solubility, **b** recovery rates (black and white bars represent the rate of silvers detected as particles and as ions, respectively), **c** average particle diameters shown in a bar chart, and **d** particle size distribution shown in a beeswarm chart were analyzed by sp-ICP-MS analysis. The results are expressed as mean ± SD (*n* = 3)

The liver homogenate was mixed with nAg100 to obtain a final Ag concentration of 100 ng/mL followed by treatment with each solubilizing reagent at 37 °C. First, we evaluated the amount of tissue residue as an indicator of tissue solubility. More than 90% of the tissue was dissolved by treatment with NaOH, TMAH, and proteinase K, while only 75% of the tissue was dissolved by HNO_3_ and HCl treatments (Fig. 2a). Considering that nearly 80% of the tissue is composed of water [19], HNO_3_, HCl, and PBS treatments were inefficient for dissolving the insoluble tissue matrix. In contrast, treatment with NaOH, TMAH, and proteinase K efficiently dissolved the insoluble matrix of tissues, indicating their suitability for accurately quantifying nAg in the tissue. Next, we analyzed the recovery rate of each particle and ion to evaluate the change in physical properties with each treatment. Sp-ICP-MS analysis showed that nAg100 was nearly completely ionized by treatment with acidic reagents (HNO_3_ and HCl) and partially ionized when treated with proteinase K. This suggested that acidic reagents and proteinase K dissolved the particles and converted them into ions. In contrast, 100 ng/mL Ag, corresponding to the initially added quantity, was detected as particles when the tissue was treated with alkaline reagents (NaOH and TMAH). Nearly no ions were detected following alkaline treatments (Fig. 2b), indicating that NaOH and TMAH maintained the nAg physical properties. Finally, we evaluated the particle diameter distribution in tissues treated with the different reagents, in order to analyze the physical properties in detail. The average particle diameter changed to 120 from 100 nm after TMAH treatment (Fig. 2c). Furthermore, the particles were broader after TMAH treatment (Fig. 2d), indicating particle aggregation. In contrast, when the tissues were treated with NaOH, the average particle diameter was close to 100 nm, corresponding to the initial particle size. This suggests that pretreatment with NaOH is the optimal condition for detecting nAg100 in mouse liver tissues.

TMAH pretreatment has been widely used for sp-ICP-MS analysis in various studies. TMAH may induce aggregation of nAg100 based on various physical properties such as viscosity and pH. Furthermore, the dielectric constant may be related to aggregation. TMAH treatment for 3 h may increase the dielectric constant caused by TMAH decomposition into trimethylamine (TMA) and methanol [20]. An increase in the dielectric constant brings the zeta potential of nAg100 which is inversely proportional to the dielectric constant, to nearly zero, resulting in the loss of electrostatic repulsion between nAg and induction of aggregation. Treatment of nAg100 with TMAH for a short time (1 min) resulted in an average particle size of approximately 100 nm (data not shown).

### Evaluation of Versatility of NaOH Pretreatment in Various Organs

**Fig. 3:**
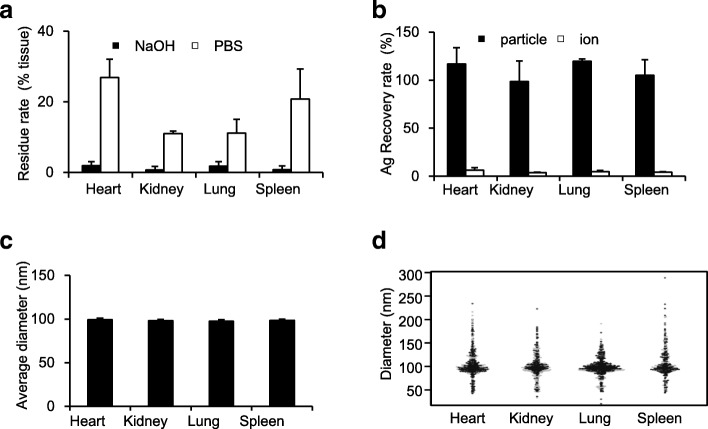
NaOH pretreatment is the optimal method for detecting nAg100 in various organs. As in Fig. 2, the heart, kidney, lung and spleen homogenates were mixed with nAg100 and incubated with NaOH solution. After 3 h, **a** residue rates (black and white bars represent the residue rates in NaOH- or PBS-treatment, respectively), **b** recovery rates (black and white bars represent the rate of Ag detected as particles and as ions, respectively), **c** average particle diameters shown in a beeswarm chart, and **d** particle size distribution shown in a beeswarm chart were analyzed by sp-ICP-MS analysis in each tissue sample. The results are expressed as mean ± SD (*n* = 3)

To evaluate the versatility of NaOH pretreatment for detecting nAg, we treated various mouse organs (heart, lung, kidney, and spleen) with NaOH and conducted sp-ICP-MS for particle detection. First, we evaluated the amount of tissue residue as an indicator of tissue solubility. More than 95% tissue solubilization was achieved by NaOH treatment (Fig. 3a). Moreover, Ag corresponding to the additive amounts was detected as particles (Fig. 3b). Although some recovery rates exceeded 100%, US Food and Drug Administration criteria state that a recover rate of 80–120% is sufficiently reliable [21]. Therefore, our analysis is reliable. Additionally, the average particle diameter of nAg detected in any organ was close to 100 nm, corresponding to the particle size of the nAg added (Fig. 3c, d). These studies suggest that NaOH pretreatment is ideal for detecting nAg not only in the mouse liver but also in the mouse heart, lung, kidney, and spleen.

Taken together, our results demonstrate that NaOH pretreatment is the optimal pretreatment strategy for the quantification and physical property analyses of nAg in animal tissues by sp-ICP-MS.

### Evaluation of sp-ICP-MS for Quantitative and Physical Property Analyses of nAg and Ag^+^ in Biological Tissues

**Fig. 4:**
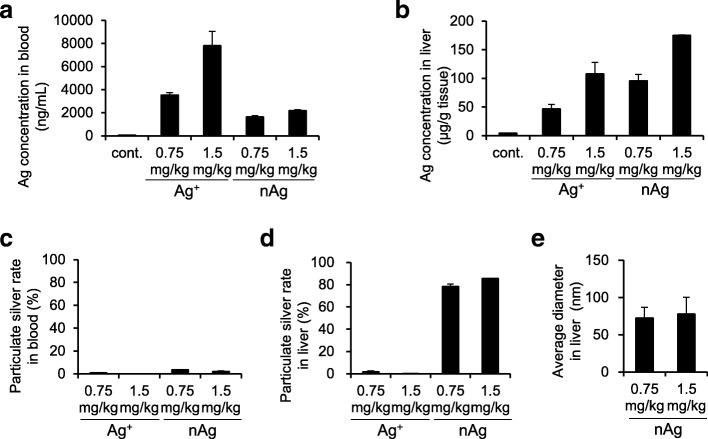
Simultaneous quantification and physical property analyses of intravenously administered nAg100 and Ag^+^. nAg100 and Ag^+^ were intravenously administrated in mice (0.75 or 1.5 mg/kg). After 24 h, their livers and blood were collected. All samples were pretreated with NaOH solution. Ag concentration in **a** blood and **b** liver were measured by ICP-MS. nAg in **c** blood and **d** liver were measured by sp-ICP-MS. The average diameter of the particles detected in the liver is shown in **e**. The results are expressed as mean ± SE (*n* = 3)

nAg ionizes in the body or that Ag^+^ particulates in mouse tissue, although the details of this process are unclear. Therefore, we evaluated the practical application of the sp-ICP-MS by analyzing both the quantity and physical properties of Ag in the mouse blood and liver after a single intravenous administration of nAg100 or Ag^+^. ICP-MS analysis showed that Ag was detected in the blood of both Ag^+^- and nAg100-treated mice (Fig. 4a). Moreover, Ag was detected in the liver of both groups (Fig. 4b). Next, we analyzed the physical properties of Ag in each sample. Because small amounts of nAg were detected in the blood of both Ag^+^- and nAg100-treated mice, most detected Ag was in the ion form (Fig. 4c). In the liver samples, approximately 80% of Ag was detected as particles in nAg100-treated mice, while a small amount of nAg was detected in Ag^+^-treated mice (Fig. 4d). Finally, we evaluated the particle size in the liver of nAg100-treated mice by sp-ICP-MS, which showed that particle sizes were approximately 80 nm (Fig. 4e). These data suggest that Ag^+^ administered into the blood hardly changed into particles, and physical properties of Ag^+^ in the blood and liver were not changed. In contrast, nAg100 administered into the blood was partially ionized; 20% of Ag in the liver and most Ag in the blood were in the ion form. As a result of partial ionization, the average diameter of the nAg in the liver tissues was smaller than that of the initially administered particles (80 vs 100 nm). Consequently, our biological sample applicable sp-ICP-MS strategy revealed that nAg100 administered into the blood was distributed as particles (approximately 80%) in the liver and as ions (approximately 95%) in the blood, while the ICP-MS method could only evaluate Ag amounts and not physical or chemical changes in the particles.

## Conclusions

We identified the optimal pretreatment conditions for sp-ICP-MS analysis of nAg in biological tissues, enabling simultaneous quantification and physical property analyses of ENPs in animal tissues. We also developed sp-ICP-MS method suitable for evaluating biological samples and demonstrated its applicability by evaluating the change in physical properties of nAg100 in the liver and blood. We also showed that the partial change from the particle form to the ionic form of nAg100 administered into the mice influenced their kinetics and distribution. This technique can be applied in the risk analysis of ENPs by evaluating the ENP exposure conditions, elucidating the biological responses to ENPs and by identifying the mechanisms underlying the responses.

## Data Availability

Data sharing is not applicable to this article as no datasets were generated or analyzed during the current study.

## Abbreviations

Ag: Silver
Ag^+^: Silver ion
DLS: Dynamic light scattering
ENPs: Engineered nanoparticles
HCl: Hydrochloric acid
HNO_3_: Nitric acid
ICP-MS: Inductively coupled plasma-mass spectrometry
nAg: Silver nanoparticles
nAg100: 100 nm nAg
nAg30: 30 nm nAg
nAg70: 70 nm nAg
NaOH: Sodium hydroxide
PBS: Phosphate-buffered saline
sp-ICP-MS: Single particle ICP-MS
TEM: Transmission electron microscope
TMAH: Tetramethylammonium hydroxide

## References

[1] Contado C (2015) Nanomaterials in consumer products: a challenging analytical problem. Front Chem. 10.3389/fchem.2015.00048 10.3389/fchem.2015.00048 PMC4527077 26301216

[2] Nel A (2007) Toxic potential of materials. Science. 10.1126/science.1114397

[3] Schäfer B, Vom BJ, Epp A, Götz M, Herzberg F, Kneuer C, Sommer Y, Tentschert J, Noll M, Günther I, Banasiak U, Böl GF, Lampen A, Luch A, Hensel A (2013) State of the art in human risk assessment of silver compounds in consumer products: a conference report on silver and nanosilver held at the BfR in 2012. Arch Toxicol. 10.1007/s00204-013-1083-8 10.1007/s00204-013-1083-8 PMC3841577 23779146

[4] Ahamed M, AlSalhi MS, Siddiqui MKJ (2010) Silver nanoparticle applications and human health. Clin Chim Acta. 10.1016/J.CCA.2010.08.016 10.1016/j.cca.2010.08.016 20719239

[5] Kettiger H, Schipanski A, Wick P, Huwyler J (2013) Engineered nanomaterial uptake and tissue distribution: from cell to organism. Int J Nanomedicine. 10.2147/IJN.S49770 10.2147/IJN.S49770 PMC3767489 24023514

[6] Miyayama T, Arai Y, Suzuki N, Hirano S (2014). Cellular distribution and behavior of metallothionein in mammalian cells following exposure to silver nanoparticles and silver ions. J Pharm Soc Japan..

[7] Loeschner K, Hadrup N, Qvortrup K, Larsen A, Gao X, Vogel U, Mortensen A, Lam HR, Larsen EH (2011) Distribution of silver in rats following 28 days of repeated oral exposure to silver nanoparticles or silver acetate. Part Fibre Toxicol. 10.1186/1743-8977-8-18 10.1186/1743-8977-8-18 PMC3123173 21631937

[8] Molleman B, Hiemstra T (2015) Surface structure of silver nanoparticles as a model for understanding the oxidative dissolution of silver ions. Langmuir. 10.1021/acs.langmuir.5b03686 10.1021/acs.langmuir.5b03686 26595806

[9] Laborda F, Jiménez-Lamana J, Bolea E, Castillo JR (2011) Selective identification, characterization and determination of dissolved silver(i) and silver nanoparticles based on single particle detection by inductively coupled plasma mass spectrometry. J Anal At Spectrom. 10.1039/c0ja00098a

[10] Pace HE, Rogers NJ, Jarolimek C, Coleman VA, Gray EP, Higgins CP, Ranville JF (2012) Single particle inductively coupled plasma-mass spectrometry: a performance evaluation and method comparison in the determination of nanoparticle size. Environ Sci Technol. 10.1021/es301787d 10.1021/es301787d 22780106

[11] Calzolai L, Gilliland D, Rossi F (2012) Measuring nanoparticles size distribution in food and consumer products: a review. Food Addit Contam - Part A Chem Anal Control Expo Risk Assess. 10.1080/19440049.2012.689777 10.1080/19440049.2012.689777 22725833

[12] Wageningen University & Research: Single Particle Calculation tool - WUR. https://www.wur.nl/en/show/Single-Particle-Calculation-tool.htm (2014) Accessed 21 Sep 2018.

[13] Laborda F, Jiménez-Lamana J, Bolea E, Castillo JR (2013) Critical considerations for the determination of nanoparticle number concentrations, size and number size distributions by single particle ICP-MS. J Anal At Spectrom. 10.1039/c3ja50100k

[14] Loeschner K, Brabrand MSJ, Sloth JJ, Larsen EH (2014) Use of alkaline or enzymatic sample pretreatment prior to characterization of gold nanoparticles in animal tissue by single-particle ICPMS. Characterisation of Nanomaterials in Biological Samples. Anal Bioanal Chem. 10.1007/s00216-013-7431-y 10.1007/s00216-013-7431-y 24154927

[15] Clegg MS, Keen CL, Lönnerdal B, Hurley LS (1981) Influence of ashing techniques on the analysis of trace elements in animal tissue - I. Wet ashing. Biol Trace Elem Res. 10.1007/BF02990451 10.1007/BF02990451 24271640

[16] Preston AM, Palacios C, Rodríguez CA, Vélez-Rodríguez RM (2011). Validation and reproducibility of a semi-quantitative food frequency questionnaire for use in Puerto Rican children. P R Health Sci J..

[17] Peters RJB, Rivera ZH, Van Bemmel G, Marvin HJP, Weigel S, Bouwmeester H (2014) Development and validation of single particle ICP-MS for sizing and quantitative determination of nano-silver in chicken meat. Characterisation of Nanomaterials in Biological Samples. Anal Bioanal Chem. 10.1007/s00216-013-7571-0 10.1007/s00216-013-7571-0 24390462

[18] Gray EPE, Coleman JGJ, Bednar AJ, Kennedy AJ, Ranville JF, Higgins CP (2013) Extraction and analysis of silver and gold nanoparticles from biological tissues using single particle inductively coupled plasma mass spectrometry. Environ Sci Technol. 10.1021/es403558c 10.1021/es403558c 24218983

[19] Pethig R, Kell D (1987). The passive electrical properties of biological systems: their significance in physiology, biophysics and biotechnology. Phys. Med. Biol..

[20] McLachlan AD (1964) Van der waals forces between an atom and a surface. Mol Phys. 10.1080/00268976300101141

[21] U.S.A. Food and Drug Administration (2009). APPENDIX 1 – ORA Validation and Verification Guidance for Human Drug Analytical Methods (ORA-LAB.5.4.5).

